# Grand Challenges in Bionics

**DOI:** 10.3389/fbioe.2013.00003

**Published:** 2013-06-18

**Authors:** Danilo De Rossi, Michael Pieroni

**Affiliations:** ^1^Research Center “E. Piaggio”, University of PisaPisa, Italy

“*After he was healed and had made himself a foot of wood, he declared himself an open enemy of the Lacedaemonians*…”^1^

“*This machine, whose 33-foot wingspan was covered in raw silk, may be powered by human muscles. Indeed, the pilot turning the crankshaft with his hands and feet make the wings flap and the machine fly*”^2^.

“Bionics” as an inter-science discipline officially dates back to 1958 when Major J. E. Steele coined the term making reference to a research program at the Wright-Patterson Air Force Base in Dayton, OH, USA. Steele (1960) used the term bionics to mean “a like-life system that copies some functions and characteristics of a natural system.”

A similar term, *biomimetics*, was coined by Schmitt (1969) in the title of his paper defined as “the study of formation, structure, or function of biologically produced substances and materials (as enzymes or silk) and biological mechanisms and processes (as protein synthesis or photosynthesis) especially for the purpose of synthesizing similar products by artificial mechanisms which mimic natural ones.”

From these original definitions, overlapping each other, different approaches and targets have emerged. In such a process a role was also played by the 1970s series “Bionic Woman” and the 1972 novel “Cyborg” of Martin Caidin, which gave “bionics” the connotation of merging biology and electronics. Also the engineering community has been sensitive to this influence and, nowadays, different meanings are used for bionics, biologically inspired design (Vincent et al., 2006; Shu et al., 2011) and integration of artificial parts into living bodies. On one hand, biologically inspired design attempts to either conceive and construct new engineering artifacts and systems, and to underpin the design principles of living system. On the other hand, the integration of artificial parts into living bodies addresses the development of prostheses and implants for body function replacement or augmentation. Both the research fields of “bionics” were addressed by two reports in *Scientific America*, entitled “Your Future with Robots” and “Your Bionic Future,” issued in 2008 and 1999, respectively. In 1999 the core of bionics focused on new body parts (implants, transplants, and prostheses) and new senses (analysis and synthesis of artificial senses and sensory substitution); however, in 2008 bioinspired robots (artificial bodies and artificial minds) found itself on the brink of bionic research.

The expected impact of bionic research on society and economics has attracted many international funding initiatives, such as *Cybernetics Hand^3^ and Bionic Ear* projects^4^, and has brought to life research consortia and networks, such as the *Convergent Science Network of Biomimetics and Biohybrid System^5^* in the EU, the European Scientific Networks for Artificial Muscles (ESNAM)^6^ and the *Bionic Vision* consortium in Australia^7^. As an additional evidence of the interest of the scientific community in the field of bionics the International Society of Bionic Engineering (ISBE)^8^ was founded in 2010 to foster the exchange of information on bionic engineering research, development, and application. Moreover, the interest of the public in bionics has been growing due to the popularization of the recent technological progress in this field. This is evidenced by the recent delivery of the “Bionic Man” built by Shadow Robot Company (London, UK) for the British Channel 4 documentary “How to Build a Bionic Man”^9^.

Lepora et al. (2013) reported and analyzed the explosive growth of publications and discoveries in the world of biomimetics of the last 15 years. They created the topography and inter-connectedness of the sub-disciplines pertaining to this research field. At a higher level of clustering, specific communities have emerged that tend to group together within the network. “Bionics” is more related to robotics (having an emphasis on biologically based control and intelligence), ethology-based robotics (having an emphasis on constructing robot hardware based on animals), and biomimetic actuators and sensors.

*Frontiers in Bioengineering and Biotechnology* has devoted two special sections to biological inspired design. The first is *Frontiers in Biomimetics*, in which the content and coverage relates more to the original definition of biomimetics focusing on the molecular, supramolecular, and cellular aspects of the mimicking effort. The second is *Frontiers in Bionics*, where focus lies on learning abstract design rules from the field of natural science, especially biology, to help in the development of new engineering concepts and to develop new engineering models and artifacts to test biological hypothesis.

Specific challenges of Frontiers in Bionics are multi-dimensional.

The first is related to body implants and prostheses, where flesh and machine meet to restore or replace lost functions of the human body (Miller, 2005; Kringelbach et al., 2007). Another aspect of this dimension may also include human augmentation in terms of sensory or motor performance. Indeed, in tackling this problem, it is crucial to consider the biocompatibility of foreign materials, toxicity, and durability. Grand challenges, which are still far from being overcome, exist in the conception and development of implantable, wearable or portable, efficient and compact energy sources. More ambitious goals aim to conceive and implement methodologies and technologies that provide workable solutions for making a connection between the central or peripheral nervous system and the artifacts in order to fully exploit the power and sophistication of brain control.

The second dimension relies on design and development of machines whose functions are very similar to biological creatures (Pfeifer et al., 2007), rather than attempt to copy the biological systems themselves. As described by Pfeifer et al. (2012), along with the development and integration of better performing technologies, such as sensors, actuators, computation, and materials, the crux resides in comprehending the principles underlying the behavior of living creatures in order to transfer these principles to the development of artifacts (Lipson and Pollack, 2000; Floreano and Mattiussi, 2008; Bongard, 2011). These principles help in overcoming the current limitations of bioinspired robotics due to the difference in materials and structures between biological creatures and artificial machines. More specifically, the major challenge lies in the real transition to soft robotics where soft body components are built out of soft actuators and sensors. Such a complex problem can only be solved through a close collaboration of soft-matter physicists, material scientists, and engineers. Finally, the increasing complexity of control may require the spontaneous development of desired skills and behaviors as a result of the body-based exploration of the environment driven by the brain (control) (Floreano and Keller, 2010). Therefore, the capacity to transfer the knowledge acquired in computational neuroscience is imperative.

Two strictly related dimensions concern *biohybrid* and *biomorphic* systems. *Biohybrid systems* exploit the combination of at least one biological and one artificial component (Reger et al., 2000; Warwick et al., 2010) in order to construct artificial system that not only mimic living ones but share similar fundamental principles. A landmark aspect of this dimension is related to mastering technologies leading to a real integration of inert and living matter to lead to a fully bionic sensory-motor system.

*Biomorphic systems* attempt to test the coherence and feasibility of principles that are thought to be at work in biological systems. Such design philosophy is aimed at constructing artificial system that perform better in the real world in terms of robustness, efficiency, and autonomy. They emulate the sensory system, architecture, and mechanisms used by animals (Sandini and Metta, 2003; Northen et al., 2008; Laschi et al., 2009), and therefore could integrate biomorphic technologies. In these systems, the key is to decode the principles offered by natural creatures in paradigms that could be treated by applying engineering design approaches. This analysis aims to overcome limitations that exist due to the difference between biological creatures and artificial artifacts in terms of design philosophy and building materials.

In addition to the development of bioinspired artifacts for achieving better performance, another dimension of interest is epistemological. The epistemological approach attempts to test and verify biology-based hypothesis by conceiving and implementing specific bioinspired machines (Webb, 2001). The goal here is to achieve adequate plausibility of these systems in order to render them representative of reality. The epistemological dimension may also comprise “artificial life” or “life-*in silico*” (Bedau et al., 2000; Westerhoff et al., 2009). Generating immaterial creatures through simulations of artificial life strives to obtain and understand the complex information processing that distinguishes living systems from the inanimate world. The additional contribution of the artificial life resides in investigating not only “life-as-we-know-it” but also “life-as-it-might-be” (Langton, 1986). A grand challenge of artificial life consists in moving from modeling and simulating to realization of concrete systems.

Progress in this inter-science area of research will only happen through increasing interaction among scientists and engineers operating in different communities through a truly multidisciplinary approach. The journal aims at providing a forum to exchange the scientific and technical results in order to foster the work on addressing the unsolved problems. The identified grand challenges should encourage to focus on ambitious goal and are not intended to limits other important issues within the different dimensions of bionics. Indeed, frequent reassessment of grand challenges will be aimed at keeping up these incentives according to the future scientific findings and the research communities’ stimuli.

## Footnotes

^1^ “Herodotus, The Histories, 9.37 narrating the events occurred to Hegesistratus of Elis,” Perseus under PhiloLogic, accessed on April 3, 2013, http://perseus.uchicago.edu/perseus-cgi/citequery3.pl?dbname=GreekTexts&getid=1&query=Hdt.%209.37

^2^ “Flying Machine (The Ornithopter), Leonardo Da Vinci inventions” accessed April 3, 2013, http://www.da-vinci-inventions.com/flying-machine.aspx

^3^ “CYBERHAND EU project ” accessed April 3, 2013, http://www-arts.sssup.it/Cyberhand/introduction/

^4^ “Bionic Ear project” last modified December 12, 2003, http://europa.eu/rapid/press-release_IP-03-1715_en.htm

^5^ “Convergent Science Network of Biomimetics and Biohybrid System” accessed April 9, 2013, http://csnetwork.eu/

^6^ “European Scientific Networks for Artificial Muscles, ESNAM” accessed April 9, 2013, http://www.esnam.eu/

^7^ “Bionic Vision consortium in Australia” accessed April 9, 2013, http://www.bionicvision.org.au/

^8^ “International Society of Bionic Engineering, ISBE” accessed April 9, 2013, www.isbe-online.org/

^9^ “How to build a Bionic Man” documentary for British Channel 4, accessed April 9, 2013, http://www.channel4.com/programmes/how-to-build-a-bionic-man
